# Polyphyllin H inhibits malignant progression of ovarian cancer in patient-derived xenograft mouse models by regulating CGN/RhoA/Rock2 axis: an experimental research

**DOI:** 10.1097/JS9.0000000000003126

**Published:** 2025-08-05

**Authors:** Guojie Chen, Donglan Yuan, Juan Li, Guangyao Mao, Yongkang Zhang, Ting Guo, Mei Lin

**Affiliations:** a Department of Clinical Medicine, Medical School of Nantong University, Nantong, Jiangsu, China; b Clinical Laboratory, The Affiliated Taizhou People’s Hospital of Nanjing Medical University, Taizhou, Jiangsu, China; c Department of Gynaecology and Obstetrics, The Affiliated Taizhou People’s Hospital of Nanjing Medical University, Taizhou, Jiangsu, China; d Graduate School, Nanjing University of Chinese Medicine, Nanjing, Jiangsu, China; e Department of Proctology, The Affiliated Taizhou People’s Hospital of Nanjing Medical University, Taizhou, Jiangsu, China; f Central Laboratory, The Affiliated Taizhou People’s Hospital of Nanjing Medical University, Taizhou, Jiangsu, China; g Department of Neurosurgery, The Affiliated Hospital of Xuzhou Medical University, Xuzhou, Jiangsu, China

**Keywords:** cingulin, ovarian cancer, PDX model, polyphyllin H, RhoA/Rock2 signaling pathway

## Abstract

**Background:**

Discovering new and effective drugs is a top priority for treating ovarian cancer, a gynecological tumor with a high mortality rate. As a major active ingredient isolated from *Paris polyphylla*, Polyphyllin H exhibits antitumor effect. However, its efficacy in ovarian cancer remains unknown. This study is designed to elucidate the antitumor activity and underlying mechanism of polyphyllin H in ovarian cancer.

**Materials and methods:**

Polyphyllin H was selected from 20 traditional Chinese medicine monomers based on its high anti-proliferative activity in CCK-8 assays performed on human ovarian cancer cell lines. The effects of polyphyllin H at different concentrations on cultured ovarian cancer cells were evaluated. Polyphyllin H was administered intragastrically to the cell line-derived xenograft (CDX) mice with ovarian cancer and the tumor inhibitory effects were assessed, and the potential anti-ovarian cancer mechanisms were predicted through protein sequencing. Subsequently, the therapeutic potential was further verified in patient-derived xenograft (PDX) mouse model.

**Results:**

The *in vitro* experiments demonstrated that polyphyllin H potently inhibited the proliferation, invasion, and migration of ovarian cancer cells while promoting cellular apoptosis. *In vivo* studies further confirmed that polyphyllin H significantly suppressed ovarian tumor growth, without inducing hepatic, renal and hematopoietic dysfunction, intestinal flora disturbance and major visceral histopathological change. Mechanistically, polyphyllin H upregulated cingulin (CGN) expression, blocking the RhoA/Rock2 signaling pathway to inhibit ovarian cancer malignant progression. Notably, polyphyllin H exerted promising antitumor efficacy in ovarian cancer PDX model, particularly demonstrating superior therapeutic effects against Her2-positive ovarian cancer.

**Conclusion:**

This study demonstrates that polyphyllin H inhibits the malignant progression of ovarian cancer via the CGN/RhoA/Rock2 pathway, without notable toxic and side effects. It provides a novel candidate drug for ovarian cancer therapy. Notably, the efficacy of polyphyllin H in ovarian cancer PDX models indicates its translational potential for clinical application.

## Introduction

Ovarian cancer is a gynecological malignant tumor with a high incidence and mortality rate^[1]^. Due to the lack of early specific indicators, most patients are diagnosed at an advanced stage^[2]^.Currently, platinum-based combination chemotherapy with paclitaxel remains the standard treatment modality, yet its efficacy in advanced-stage patients is suboptimal. Approximately 70% of patients experience relapse within three years, while chemotherapy frequently induces severe complications including bone marrow suppression, hepatorenal dysfunction, and intestinal flora dysbiosis, which substantially impact patient morbidity^[3]^. Consequently, there is a clinical imperative to identify novel, efficacious, and safe therapeutic agents to improve prognosis for ovarian cancer patients.

In recent years, extensive research on plant-derived anticancer compounds has been conducted as to identify safe and effective alternatives to conventional, drug-based treatments. Several bioactive components of the dried rhizome of *Paris polyphylla*, a medicinal herb of the Liliaceae family endemic to the Himalayan region, have demonstrated antitumor, anti-inflammatory, antioxidant, and analgesic functions^[4]^. While this evidence prompted increasing interest in characterizing the active ingredients of *Paris polyphylla* and studying their therapeutic effects, the antitumor activity of polyphyllin H, one of the major bioactive saponin constituents of this medicinal herb, in highly malignant ovarian cancer remains unknown. In addition, similarly to other structurally complex traditional Chinese medicine (TCM) monomers, whether polyphyllin H can exert anticancer effects in different patients with distinct disease state and tumor features, while also offering a higher safety profile than current clinically used therapeutic drugs, are important issues that need to be addressed experimentally.

In preclinical studies of ovarian cancer, the cell line-derived xenograft (CDX) model allows for relatively simple and rapid examination of tumor characteristics and evaluation of the therapeutic potential of candidate drugs. However, these models lack the intrinsic heterogeneity characteristic of human tumors^[5,6]^, and thus their clinical translational significance is largely limited. In recent years, patient-derived xenograft (PDX) animal models, which arguably improve the translational value of anticancer research, have been constructed by transplanting cancer tissue from patients into immunodeficient (e.g., NGS) mice^[7]^. Compared with CDX model, PDX model retains the genetic characteristics of the original patient’s tumor and simultaneously satisfy three important criteria for effective disease models, namely face validity, target validity, and predictive validity^[8,9]^. Such models are therefore particularly reliable in predicting the clinical efficacy of candidate drugs^[10]^.

This study is aimed to evaluate the therapeutic effects of polyphyllin H in ovarian cancer, study the underlying antitumor mechanism, and examine its safety and translational significance. We used tandem mass tags proteomics sequencing and analysis to determine the signaling pathways regulated by polyphyllin H and verified these findings in human ovarian cancer cell lines and the corresponding CDX mouse models. In addition, we generated four PDX mouse models derived from patients with ovarian cancer to explore the therapeutic effect of polyphyllin H, and achieved desirable results. Our findings lay a foundation for further studies evaluating the antitumor action of polyphyllin H and its application in ovarian cancer treatment.

## Materials and methods

### Cell culture, compounds, and cell transfection

Ovarian cancer cell lines ES-2 and SKOV-3 with STR verification were purchased from Procell Life Science & Technology Co., Ltd. (China) and cultured in Dulbecco’s modified Eagle medium (DMEM) containing 10% fetal bovine serum (FBS). Polyphyllin H (purity ≥ 98.0%; CAS: 81917-50-2) was purchased from Pusi Biotech Co., Ltd. (China), and lysophosphatidic acid (LPA) (purity ≥ 99.0%; CAS: 52603-03-9) was purchased from MedChemExpress (China). To silence cingulin (CGN), ES-2 and SKOV-3 cells were transfected with lentiviral sh-CGN plasmids, chemically synthesized by Shanghai GenePharma Co., Ltd (China). The sequences of sh-CGN as follows: 5ʹ-GATCCCCGCTGAAGAAGATGAAGATTTCAAGAGAATCTTCATCTTCTTCAGCTTTTTGGAAA-3ʹ.

**HIGHLIGHTS**

- Polyphyllin H with anti-ovarian cancer activity was screened from 20 traditional Chinese medicine monomers, providing a new drug candidate for ovarian cancer treatment.
- The anti-tumor mechanism of Polyphyllin H was studied in depth through various means such as proteomic analysis, cell experiments, and animal experiments.
- The cell line-derived xenograft (CDX) model and the patient-derived xenograft (PDX) model were used for research. In particular, the PDX model can better reflect the heterogeneity of human tumors and improve the clinical translational value of the research results.
- Evaluated comprehensively polyphyllin H safety *in vivo*, including the effects on the liver, kidney, hematopoietic system, and intestinal flora, providing an important basis for its clinical application.

### Cell viability assay

To perform cell viability assays, control and sh-CGN-transfected ES-2 and SKOV-3 cells were exposed to CCK-8 reagent (APExBIO Corporation, USA) for 1 h, and absorbance was measured using a microplate reader (BioTek, USA).

### Colony formation assay

Control and sh-CGN-transfected ES-2 and SKOV-3 cells were stimulated with different concentrations of polyphyllin H and LPA, and 1000 cells per well were evenly spread in a 6-well plate and then cultured at 37°C for 21 days. The cultures were then fixed with 4% paraformaldehyde, and stained with 0.05% crystal violet. Colony numbers were counted using a microscope (Nikon, Japan).

### Cell migration and invasion assays

To assess cell migratory capacity, a linear wound was created across near-confluent ES-2 and SKOV-3 cell monolayers. Images were captured at 0 h and 24 h post-wounding to measure wound closure for quantifying cell migration. Cell invasion capacity was detected by Transwell assays. 1 × 10^5^ ES-2 and SKOV-3 cells were respectively added to the upper chamber of Matrigel-coated Transwell inserts. Medium with 20% FBS was added to the lower chamber. After 3 days, non-migrated cells were removed, and migrated cells were fixed, stained, counted, and photographed under an inverted microscope (Nikon, Japan).

### Apoptosis assay

Cell apoptosis was analyzed using flow cytometry. ES-2 and SKOV-3 cells were washed three times with PBS, stained with FITC and PI solutions in the dark at room temperature for 20 min, and evaluated using flow cytometry.

### Western blotting

Total proteins were extracted from ES-2 and SKOV-3 cells using RIPA lysis buffer with phenylmethylsulfonyl fluoride (Beyotime, USA). After quantification with a BCA protein assay kit, equal amounts of proteins were separated by 10% SDS-PAGE and transferred onto PVDF membranes. After blocking in 5% skim milk powder for 2 hours at room temperature, the membranes were incubated with primary antibodies against cingulin (PA5-55661; Thermo-Fisher, USA), RhoA (MA1-134; SAB, USA), Rock2 (PA5-77830; Thermo-Fisher, USA), or GAPDH (ab8245; Abcam, UK) overnight at 4°C. After washing and incubation with a secondary antibody conjugated with HRP for 2 h at room temperature, signals were visualized using Pierce™ ECL Protein Blot Substrate (Thermo Fisher Scientific, USA).

### Ovarian cancer CDX mouse model establishment and Polyphyllin H intervention

4 to 6-week-old nude mice were selected, and ovarian cancer cell line of ES-2 and SKOV-3 (1.5 × 10^6^) was subcutaneously inoculated into the right lower back to establish two CDX mouse models of ovarian cancer, respectively. When the tumor volume reached 100 mm^3^, the mice were randomly divided into two groups: control group and Polyphyllin H group, with 3 mice in each group. Polyphyllin H group mice were gavaged with Polyphyllin H (5 mg/g) every 4 days, while control group mice were gavaged with normal saline. Following the intervention, tumor long and short diameters were measured with vernier caliper every 4 days. Tumor volume was calculated using the formula “tumor volume (mm^3^) = 0.5 × (long diameter × short diameter^2^),” and the dynamic changes were documented. Additionally, the general status of each mouse was monitored throughout the experiment.

Thirty days later, the mice were sacrificed. Tumor tissues were collected for proteomic sequencing, while blood samples were obtained for routine blood tests including red blood cell (RBC), white blood cell (WBC), hemoglobin (Hb) and platelet (PLT) to evaluate bone marrow hematopoietic function and blood biochemistry analysis including serum alanine aminotransferase (ALT), aspartate aminotransferase (AST), alkaline phosphatase (ALP), total bilirubin (TBIL) and urea nitrogen (BUN) to evaluate hepatic and renal functions. H&E staining was performed on major visceral organs to observe histopathological changes. 16S RNA sequencing was conducted on mouse feces to assess the drug effects on intestinal flora.

### Ovarian cancer PDX mouse model establishment and Polyphyllin H treatment

Tumor tissue blocks from four ovarian cancer patients in Taizhou People’s Hospital were collected. The samples were placed in DMEM with 10% FBS, transported to the lab and kept on ice until implanted into NSG mice. Four cases of PDX model were established by subcutaneously implanting a 1-mm^3^ fresh or freeze-thawed patient tumor tissue block immersed in Matrigel and sealing with surgical glue. Tumor tissue block or single tumor cell suspension was frozen and saved during each passage. Successful transplantation was defined as the PDX being able to continuously transplant. The mice initially inoculated in each case were numbered as F0, and F4 generation mice were selected. This work was conducted in accordance with the ARRIVE guidelines^[11]^. Similar to the above CDX mice, we treated each of the four PDX mouse with polyphyllin H, evaluated the antitumor efficacy, and investigated the molecular mechanism. For toxicological assessment, in addition to biochemical assay to evaluate hepatic and renal functions and blood cell analyze to assess bone marrow hematopoietic function, histopathology of major visceral organs was examined via HE staining.

### Statistical analysis

Data from three independent experiments were expressed as mean ± standard deviation (mean ± s). Paired or unpaired Student’s t-test analyzed differences between two groups. One-way ANOVA and Tukey’s test evaluated differences among multiple groups. Pearson’s correlation coefficient tested correlation between gene expression data. *P* <0.05 was considered significant.

## Results

### In vitro screening of anti-ovarian cancer TCM monomers

**Figure 1.**
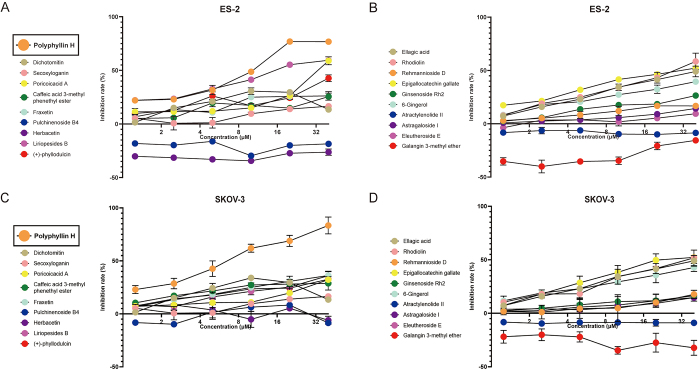
screening of TCM monomers against ovarian cancer. (A–B) The inhibitory effects of 20 TCM monomers on the ES-2 cell line were screened by the CCK-8 assay respectively. (C–D) The inhibitory effects of 20 TCM monomers on the SKOV-3 cell line were screened by the CCK-8 assay respectively.

To screen candidate TCM monomers for treating ovarian cancer, 20 TCM monomers reported in the literature to potentially have anti-ovarian cancer efficacy were tested in human ES-2 and SKOV3 ovarian cancer cells. After exposure to different concentrations of these monomers for 48 h, CCK-8 cell viability assays showed that eight of them had an inhibitory effect on ES-2 cells (Fig. 1A, B), whereas six had an inhibitory effect on SKOV-3 cells (Fig. 1C, D). Among the monomers tested, polyphyllin H had a relatively strong growth inhibitory activity in both ovarian cancer cell lines. Therefore, the anti-ovarian cancer properties of polyphyllin H were further studied.

### Polyphyllin H inhibits the malignant progression of ovarian cancer cells

**Figure 2.**
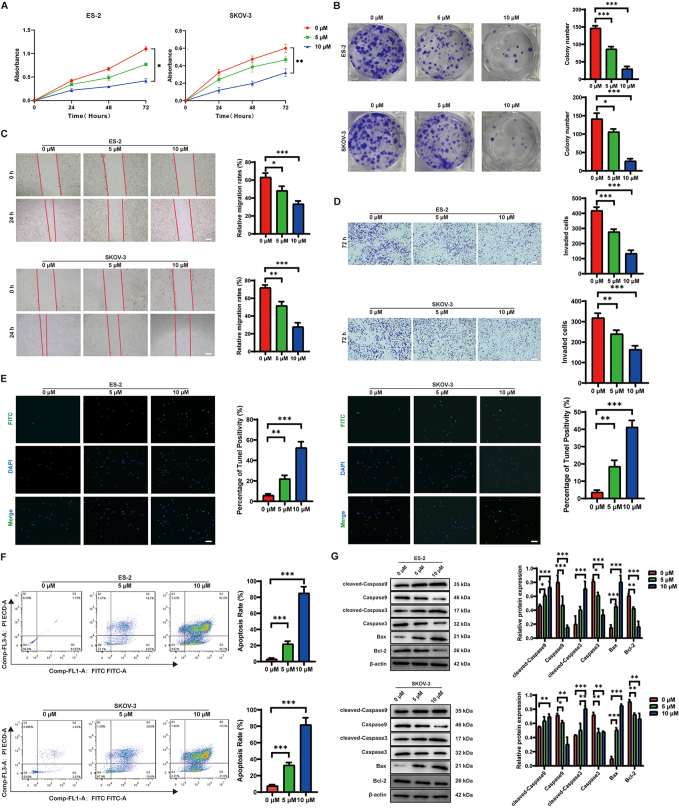
Polyphyllin H inhibits the malignant progression of ovarian cancer cells. (A) The effect of different concentrations of polyphyllin H on the proliferation level of ovarian cancer cells was detected by the CCK-8 assay. (B) The effect of different concentration of polyphyllin H on the proliferation level of ovarian cancer cells was detected by the cell colony formation assay. (C) The effect of different concentrations of polyphyllin H on the migration ability of ovarian cancer cells was detected by the wound healing assays. (D) The effect of different concentrations of polyphyllin H on the invasion ability of ovarian cancer cells was detected by the Tanswell assays. (E) The effect of different concentrations of polyphyllin H on the apoptosis of ovarian cancer cells was detected by the TUNEL assays. (F) The effect of different concentrations of polyphyllin H on the apoptosis of ovarian cancer cells was detected by flow cytometry assays. (G) The effect of different concentrations of polyphyllin H intervention on the expression level of apoptosis proteins in ovarian cancer cells was detected by Western bolt. ***P < 0.001, **P < 0.01, *P < 0.05.

To further verify the inhibitory activity of polyphyllin H on the malignant progression of ovarian cancer, the proliferation ability of ES-2 and SKOV-3 cells treated with different concentrations of polyphyllin H was quantified using CCK-8 and colony formation assays. The results showed that polyphyllin H inhibited both proliferation (Fig. 2A) and colony forming ability (Fig. 2B) of ovarian cancer cells in a time- and dose-dependent manner. The migration and invasion abilities of tumor cells are important indicators of malignant tumor progression. Using wound healing and Transwell assays to respectively assess migration and invasion potential, we confirmed that polyphyllin H inhibited migration (Fig. 2C) and invasion (Fig. 2D) of ES-2 and SKOV-3 cells in a dose-dependent manner.

To evaluate whether polyphyllin H exerts also pro-apoptotic effects, AnnexinV/PI flow cytometry and TUNEL assay were performed in ES-2 and SKOV-3 cells treated with polyphyllin H at different concentration . The results showed that as the concentration of polyphyllin H increased, the number of apoptotic cells also increased (Fig. 2E, F). To further verify this effect, western blotting was used to detect apoptosis-related proteins. The results showed that as polyphyllin H concentration increased, cleaved-Caspase-9, cleaved-Caspase-3, and Bax expression levels were increased, whereas Caspase-9, Caspase-3, and Bcl-2 expression levels were decreased (Fig. 2G). These data demonstrated that polyphyllin H exerts concentration-dependent inhibitory effects on the malignant progression of ovarian cancer cell lines by reducing their proliferation, migration, and invasion, while simultaneously promoting cell apoptosis .

### Polyphyllin H inhibits ovarian cancer cell growth in CDX mouse models, with no toxic and side effects

**Figure 3.**
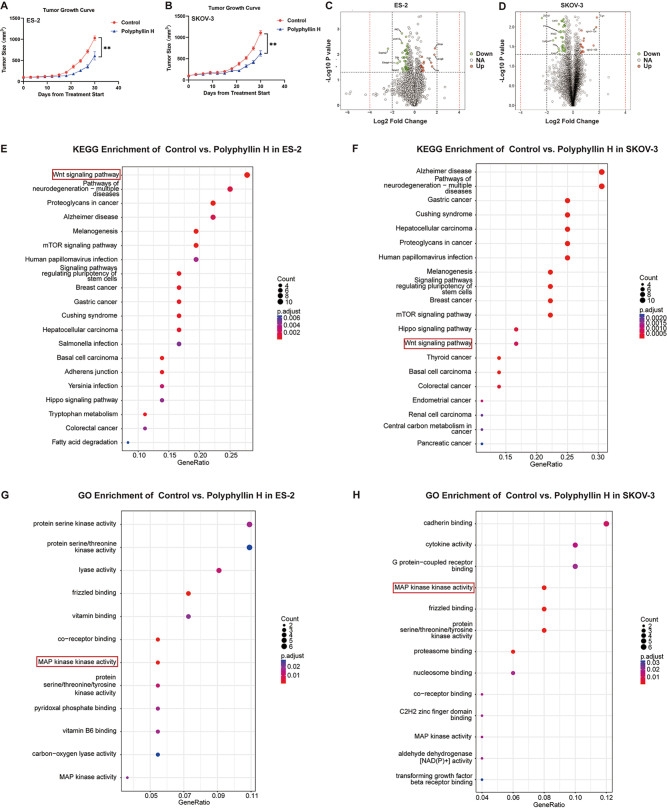
The tumor inhibitory effect of polyphyllin H in the CDX ovarian cancer model. (A) The effect of polyphyllin H on tumor volume in the CDX model constructed by ES-2. (B) The effect of polyphyllin H on tumor volume in the CDX model constructed by SKOV-3. (C) Volcanic map of differential proteins without polyphyllin H in the CDX model constructed by ES-2. (D) Volcanic map of differential proteins without polyphyllin H in the CDX model constructed by SKOV-3. (E) KEGG enrichment analysis of differential proteins in the CDX model constructed by ES-2. (F) KEGG enrichment analysis of differential proteins in the CDX model constructed by SKOV-3. (G) GO enrichment analysis of differential proteins in the CDX model constructed by ES-2. (H) GO enrichment analysis of differential proteins in the CDX model constructed by SKOV-3. **P < 0.01.

Based on the above *in vitro* results, using ovarian cancer CDX mouse models established with ES-2 and SKOV-3 cells, we next tested the safety and anticancer efficacy of polyphyllin H *in vivo*. The results showed that after intragastric administration of polyphyllin H, tumor growth was significantly inhibited (Tumor Inhibition Rate: ES-2 = 45.92 ± 9.26%, SKOV-3 = 57.29 ± 4.73%) (Fig. 3A, B), with no obvious effect on body weight (Supplementary Digital Content Figure 1A–B, available at: http://links.lww.com/JS9/E808).

In parallel, the safety of polyphyllin H was assessed by evaluating liver, kidney, and bone marrow hematopoietic functions through serum biochemistry and blood routine tests. After polyphyllin H treatment, serum ALT, AST, ALP, TBIL, BUN (Supplementary Digital Content Figure 1C–G, available at: http://links.lww.com/JS9/E808) and blood RBC, WBC, Hb and PLT of the mice (Supplementary Digital Content Figure 1H–K, available at: http://links.lww.com/JS9/E808) did not differ significantly from that of the corresponding control mice (*P* > 0.05), indicating no damage to hepatic function, renal function and bone marrow hematopoietic function.

16S rRNA sequencing can accurately evaluate compositional changes in intestinal flora. We performed 16S rRNA sequencing of the intestinal flora of CDX mice, and found that there was no significant difference in bacterial species distribution in CDX mice before and after polyphyllin H gavage treatment (Supplementary Digital Content Figure 2A–C, available at: http://links.lww.com/JS9/E808). This was confirmed through subsequent ɑ-diversity analysis (Supplementary Digital Content Figure 2D–F, available at: http://links.lww.com/JS9/E808) and β-diversity analysis (Supplementary Digital Content Figure 2G–H, available at: http://links.lww.com/JS9/E808), which revealed no significant difference in species richness, evenness, and gut flora composition.

In view of no detrimental effects on liver and kidney function, bone marrow hematopoiesis, and intestinal flora homeostasis, it was concluded that oral or intragastric administration of polyphyllin H is safe.

### Proteomic analysis in CDX models

We next explored the mechanism underlying the antitumor activity of polyphyllin H in ovarian cancer through proteomic analysis of implanted tumors. Using |log fold change| >1 and adjusted *P* value <0.05 as screening thresholds, a total of 99 differentially expressed proteins were detected between ES-2 tumors from polyphyllin H-treated and untreated (control) mice. Compared with the control group, there were 53 upregulated and 46 downregulated proteins in the polyphyllin H group of mice (Fig. 3C). Under the same screening conditions, in the CDX model constructed with SKOV–3 cells, a total of 124 proteins were retrieved. Compared with the control group, there were 47 upregulated and 77 downregulated proteins in the polyphyllin H group (Fig. 3D). An intersection analysis of the proteins obtained from the two CDX models revealed that CGN was commonly upregulated in polyphyllin H-treated mice. On KEGG and GO enrichment analyses, both groups of proteins were found to be enriched in “KEGG: Wnt signaling pathway,” “GO: MAP kinase kinase activity,” and various tumor-related pathways (Fig. 3E-H). These data suggest that CGN may be involved in the mechanism by which polyphyllin H inhibits ovarian cancer growth.

### Polyphyllin H inhibits ovarian cancer progression by upregulating CGN

**Figure 4.**
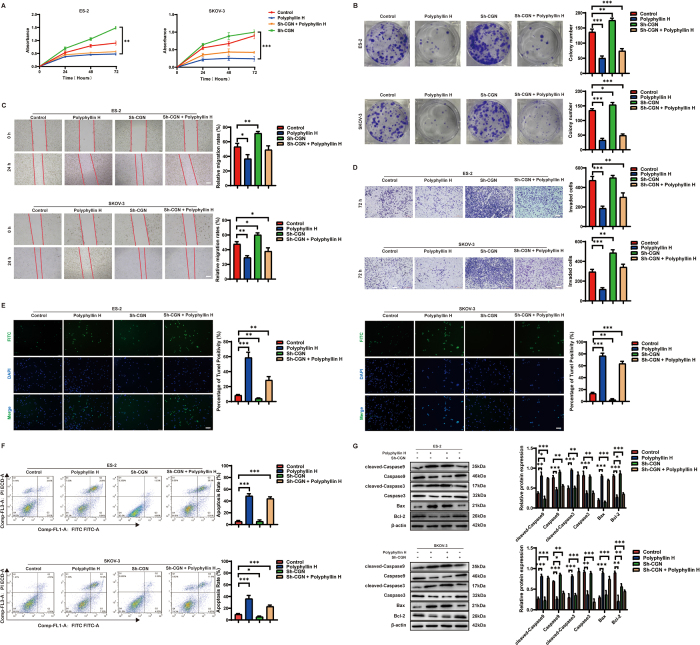
Polyphyllin H inhibits ovarian cancer by up-regulating CGN. (A) The effect of polyphyllin H on the proliferation level of ovarian cancer cells without knockdown/knockdown of CGN was detected by the CCK-8 assays. (B) The effect of polyphyllin H on the proliferation level of ovarian cancer cells without knockdown/knockdown of CGN was detected by the cell colony formation assay. (C) The effect of polyphyllin H on the migration ability of ovarian cancer cells without knockdown/knockdown of CGN was detected by the wound healing assays. (D) The effect of polyphyllin H on the invasion ability of ovarian cancer cells without knockdown/knockdown of CGN was detected by the Tanswell assays. (E) The effect of polyphyllin H on the apoptosis of ovarian cancer cells without knockdown/knockdown of CGN was detected by the TUNEL assays. (F) The effect of polyphyllin H on the apoptosis of ovarian cancer cells without knockdown/knockdown of CGN was detected by flow cytometry assays. (G) The effect of polyphyllin H on the expression level of apoptosis proteins in ovarian cancer cells without knockdown/knockdown of CGN was detected by Western bolt. ***P < 0.001, **P < 0.01, *P < 0.05.

CGN is a cytoskeletal adaptor protein located on the cytoplasmic surface of epithelial tight junctions^[12]^. It interacts with several tight junction-related proteins and participates in the malignant progression of various tumors^[13]^. To explore whether polyphyllin H inhibited the malignant progression of ovarian cancer cells by affecting the expression level of CGN, proliferation, migration, invasion, and apoptosis were assessed after knocking down CGN. After shRNA-mediated CGN knockdown, the proliferation rates of ES-2 and SKOV-3 cells were increased. However, this effect was significantly inhibited after polyphyllin H exposure (Fig. 4A, B). Similarly, CGN silencing enhanced the migratory and invasive abilities of ovarian cancer cells, and polyphyllin H treatment reversed these effects (Fig. 4C, D). Moreover, after knocking down CGN, apoptosis was reduced in both cell lines, and this effect was also reversed by polyphyllin H (Fig. 4E-G). These findings indicate that CGN upregulation mediates the inhibitory action of polyphyllin H on ovarian cancer.

### Polyphyllin H inhibits malignant progression of ovarian cancer through the CGN/RhoA/Rock2 axis

**Figure 5.**
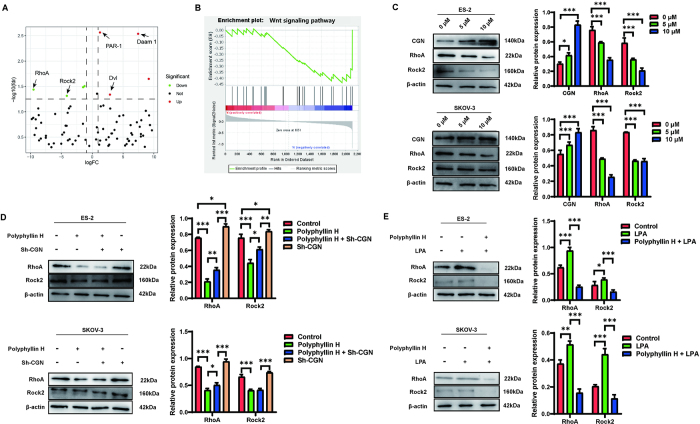
High expression of CGN inhibits ovarian cancer by inhibiting the RhoA/Rock2 pathway. (A) Volcanic map of differential proteins with high/low expression of CGN based on the TCGA-OV database. (B) Pathway correlation analysis of differential proteins based on the GSEA database. (C) The effect of different concentrations of polyphyllin H on the expression level of CGN/RhoA/Rock2 pathway proteins in ovarian cancer cells was detected by Western bolt. (D) The effect of polyphyllin H on the expression level of RhoA/Rock2 pathway proteins in ovarian cancer cells without knockdown/knockdown of CGN was detected by Western bolt. (E) The effect of polyphyllin H on the expression level of RhoA/Rock2 pathway proteins in ovarian cancer cells after LPA activation was detected by Western bolt. ***P < 0.001, **P < 0.01, *P < 0.05.

To explore the signaling pathways responsible for the decrease in ovarian cancer malignancy caused by CGN upregulation, differential gene expression analysis was initially conducted on the TCGA database. The results showed that the expression of both Ras homolog family member A (RhoA) and Rho-associated coiled-coil containing protein kinase 2 (Rock2) was significantly lower in ovarian cancer samples with high, rather than low, CGN expression (Fig. 5A). Meanwhile, gene set enrichment analysis results showed that CGN had a significant negative correlation with the Wnt pathway (Fig. 5B).

GTPase RhoA is an important component of the Wnt signaling pathway and regulates MAP kinase kinase activity. RhoA activates effector serine/threonine kinases, i.e., Rock1/Rock2, to regulate cytoskeleton dynamics, playing also an important role in tumorigenesis. To evaluate parallel changes in CGN, RhoA, and Rock2 expression induced by polyphyllin H, western blot analysis was performed on ES-2 and SKOV-3 cells. Results showed that in both cell lines and with increasing concentrations of polyphyllin H, CGN expression increased whereas RhoA and Rock2 expression decreased (Fig. 5C). In turn, when CGN was knocked down, the expression of RhoA/Rock2 increased significantly, and this upregulation was partially inhibited by polyphyllin H (Fig. 5D).

Lysophosphatidic acid (LPA) acts as a RhoA/Rock2 pathway agonist by activating G protein-coupled receptors upstream of RhoA. We thus conducted rescue experiments by exposing ES-2 and SKOV-3 cells to LPA. After LPA treatment, the expression of RhoA and Rock2 increased significantly, and the RhoA/Rock2 pathway was activated. Conversely, when polyphyllin H was added along with LPA, the expression of RhoA and Rock2 decreased significantly, and the RhoA/Rock2 pathway was inhibited (Fig. 5E). We conclude that polyphyllin H inhibits the malignant progression of ovarian cancer cells by upregulating CGN and inhibiting the RhoA/Rock2 signaling pathway.

### LPA reverses the inhibitory effect of polyphyllin H on ovarian cancer

**Figure 6.**
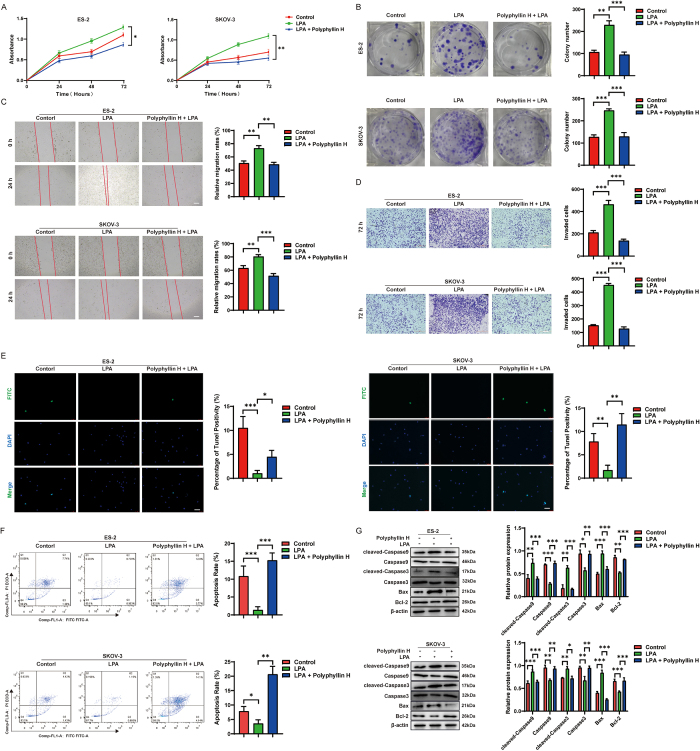
The recovery experiment of RhoA/Rock2 pathway agonists on the inhibitory effect of polyphyllin H on the malignant process of ovarian cancer cells. (A) The effect of polyphyllin H on the proliferation level of ovarian cancer cells after LPA activation was detected by the CCK-8 assays. (B) The effect of polyphyllin H on the proliferation level of ovarian cancer cells after LPA activation was detected by the cell cloning formation assay. (C) The effect of polyphyllin H on the migration ability of ovarian cancer cells after LPA activation was detected by the wound healing assays. (D) The effect of polyphyllin H on the invasion ability of ovarian cancer cells after LPA activation was detected by the Tanswell assays. (E) The effect of polyphyllin H on the apoptosis of ovarian cancer cells after LPA activation was detected by the TUNEL assays. (F) The effect of polyphyllin H on the apoptosis of ovarian cancer cells after LPA activation was detected by flow cytometry assays. (G) The effect of polyphyllin H on the expression level of apoptosis proteins in ovarian cancer cells after LPA activation was detected by Western bolt. ***P < 0.001, **P < 0.01, *P < 0.05.

The RhoA/Rock2 pathway participates in tumor cell proliferation, migration, and invasion^[14,15]^. After confirming that polyphyllin H inhibits the RhoA/Rock2 pathway through upregulating CGN, we used LPA to verify whether RhoA/Rock2 signaling activation would promote the proliferation, migration, and invasion of ovarian cancer cells. After LPA exposure, the proliferation rates of ES-2 and SKOV-3 cells were increased, and this effect was significantly inhibited by polyphyllin H (Fig. 6A, B). Further supporting a pro-tumorigenic role of the RhoA/Rock2 axis in ovarian cancer, wound healing and Transwell assays showed that LPA exposure reduced the inhibitory effect of polyphyllin H on the migration and invasion of ES-2 and SKOV-3 cells (Fig. 6C, D). In addition, we found that co-incubation with LPA reversed the pro-apoptotic effect of polyphyllin H in these cells (Fig. 6E-G). These results further confirm that polyphyllin H restrains ovarian cancer progression by inhibiting the RhoA/Rock2 pathway.

### Polyphyllin H inhibits ovarian cancer growth in PDX mouse models

**Figure 7.**
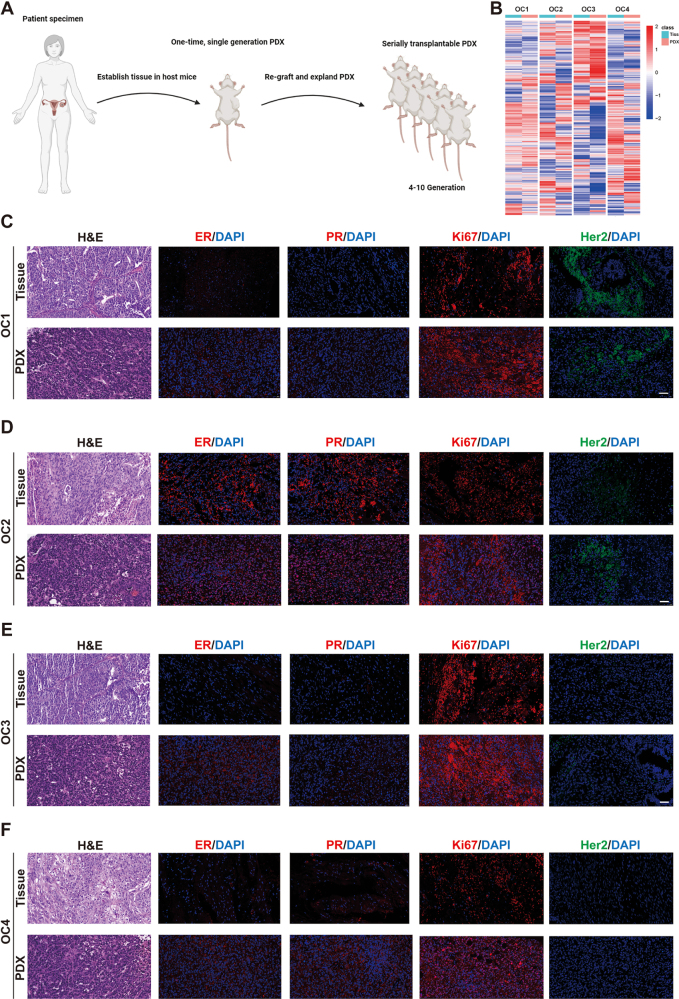
Construction of the PDX model. (A) Schematic diagram of PDX model construction. (B) Heat map of differential proteins in patient ovarian cancer tissue and PDX model tumor tissue. (C–F) H&E and Immunofluorescence staining of ovarian cancer-specific proteins in ovarian cancer tissues and PDX model tumor tissues of 4 patients.

The above research using cultured cells and CDX models highlighted the anticancer effects of polyphyllin H in ovarian cancer and its underlying mechanism. Given that animal PDX model, generated from patient-derived tumor tissues, is more likely to offer clinical relevance and facilitate personalized therapy due to their preservation of tumor interpatient heterogeneity, we constructed four PDX mouse models, termed PDX.OC1-4 (Fig. 7A), from samples collected from four patients with ovarian cancer, to further study the translational potential of polyphyllin H for ovarian cancer treatment. These models were validated by proteomic analysis (Fig. 7B) and immunohistochemical analysis. The results demonstrated successful construction of the ovarian cancer PDX model. Notably, immunohistochemical staining revealed that the expression profiles of four key ovarian cancer markers (ER, PR, Her2, and Ki-67) in all four PDX mouse models were highly consistent with those observed in the tumor tissues of their respective donor patients (Fig. 7C-F). Among them, Her2 was positive in PDX.OC1 and PDX.OC2 (Her2^+^), and negative in PDX.OC3 and PDX.OC4 (Her2^-^). Subsequently, the PDX mouse models were used to investigate the anti-ovarian cancer actions of polyphyllin H.

**Figure 8.**
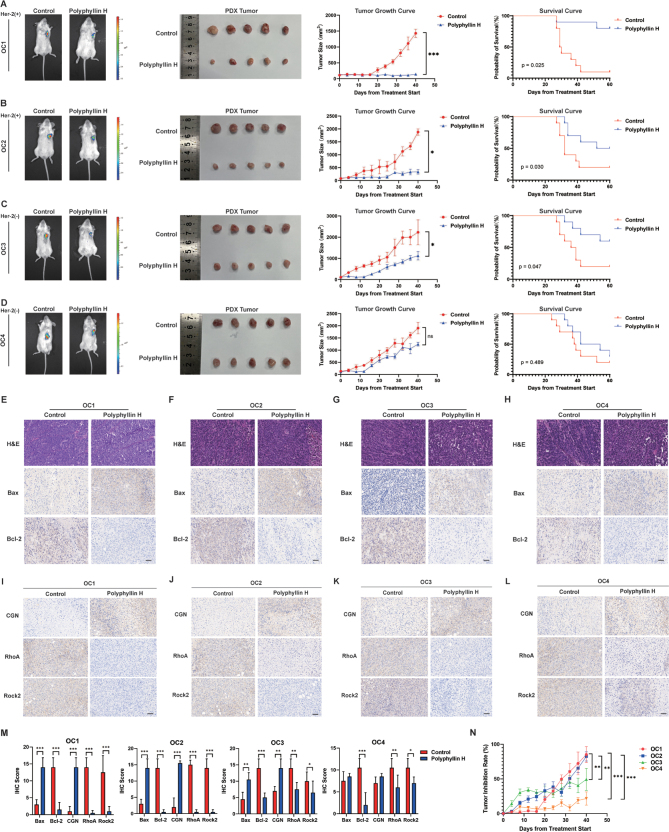
Verification of the inhibitory effect of polyphyllin H on ovarian cancer in the PDX model. (A–D) The effect of polyphyllin H on tumor size and survival time in the PDX model. (E–H) The histopathology and the effect of polyphyllin H on the expression level of RhoA/Rock2 pathway proteins in the PDX model. (I–L) The effect of polyphyllin H on the expression level of apoptosis proteins in the PDX model. (M) Statistical graph of immunohistochemistry. (N) Inter-group comparison of tumor suppression rate in PDX model.***P < 0.001, *P < 0.05.

After gavage treatment with polyphyllin H, PDX.OC1, PDX.OC2 and PDX.OC3 tumors showed significant growth inhibition (Tumor Inhibition Rate: OC1 = 85.28 ± 11.45%, OC2 = 82.43 ± 6.59%, OC3 = 49.39 ± 15.92%), and a significantly longer total survival period was recorded for these mice compared to the corresponding control group (OC1: *P* = 0.025, OC2: *P* = 0.030, and OC3: *P* = 0.047) (Fig. 8A-C). In the PDX.OC4 group, a trend toward tumor inhibition was observed after gavage administration of Polyphyllin H, but it was without significance compared to the corresponding control group (Tumor Inhibition Rate: OC4 = 21.74 ± 9.52%). Similarly, no significant difference in the total survival period (*P* = 0.489) was recorded among PDX.OC4 mice (Fig. 8D). Nevertheless, H&E staining revealed that the tumor cells in the Polyphyllin H group exhibited typical features of karyopyknosis and karyorrhexis, indicating that the apoptosis of tumor cells increased after polyphylin H intervention. Meanwhile, ex-vivo immunostaining of Bax (OC1: *P* < 0.001, OC2: *P* < 0.001, OC3: *P* = 0.006, and OC4: *P* = 0.382) and Bcl-2 (OC1: *P* < 0.001, OC2: *P* < 0.001, OC3: *P* < 0.001, and OC4: *P* < 0.001) showed increased tumor cell apoptosis in all four PDX models treated with polyphyllin H (Fig. 8E-H&M). We next analyzed, as done above *in vitro*, the expression of CGN, RhoA, and Rock2 in the PDX models, and verified that polyphyllin H increased the expression of CGN (OC1: *P* < 0.001, OC2: *P* < 0.001, OC3: *P* = 0.008, and OC4: *P* = 0.0967) while inhibiting the expression of RhoA (OC1: *P* < 0.001, OC2: *P* < 0.001, OC3: *P* = 0.003, and OC4: *P* = 0.008) and Rock2 (OC1: *P* < 0.001, OC2: *P* < 0.001, OC3: *P* = 0.042, and OC4: *P* < 0.028) (Fig. 8I-M). Notably, the growth inhibition in Her2^+^ PDX mice (PDX.OC1 and PDX.OC2) was both higher than that in Her2^−^ PDX mice (PDX.OC3 and PDX.OC4) (OC1 vs. OC3: *P* = 0.004, OC1 vs. OC4: *P* < 0.001, OC2 vs. OC3: *P* = 0.003, OC2 vs. OC4: *P* < 0.001) (Fig. 8N).

Viscera histology assess by H&E staining showed no histopathological changes in heart, liver, spleen, lung, kidney, and intestine (Supplementary Digital Content Figure 3, available at: http://links.lww.com/JS9/E808). Serum biochemistry (Supplementary Digital Content Figure 4A–E, available at: http://links.lww.com/JS9/E808) and blood routine tests (Supplementary Digital Content Figure 4F–I, available at: http://links.lww.com/JS9/E808) verified no dysfunction of liver, kidney and bone marrow hematopoiesis, further proving the safety of oral or gavage administration of polyphyllin H.

## Discussion

Ovarian cancer is a gynecological malignant tumor with rapid proliferation, easy recurrence, and a high mortality rate, for which few treatment drugs are available. In recent years, TCM has attracted increasing attention in cancer research. Among the many TCM compounds so far tested, saponin monomers have shown good antitumor performance^[16]^. Polyphyllin H is a steroidal saponin present in *Paris polyphylla*, a medicinal plant. Through molecular analyses of subcutaneous xenografts established using human ovarian cancer cells in NSG mice, we found that polyphyllin H inhibited ovarian cancer by upregulating CGN protein levels and inhibiting the RhoA/Rock2 pathway. More importantly, the translational potential of polyphyllin H for ovarian cancer treatment was asserted in four PDX models of ovarian cancer, which more accurately reproduce the complexity and heterogeneity of human tumors.

Our proteomic analysis revealed CGN as a key polyphyllin H-regulated gene in ovarian cancer cells. CGN and paracingulin (CGNL1) are key components of the apical junction complex of vertebrate epithelial cells and endothelial cells^[17]^. Both proteins play a regulatory role in epithelial barrier function and thus influence many pathological processes, including inflammation and tumorigenesis^[18]^. In recent years, many studies have reported the involvement of CGN in the occurrence and development of tumors^[19,20]^.Rho GTPases are important molecular switches that cycle between inactive (GDP-bound) and active (GTP-bound) states. CGN was shown to affect the activity and distribution of RhoA by interacting with RhoA guanidine exchange factors to regulate cell adhesion, polarity, and formation of cell-to-cell connections. In this way, CGN participates in the reorganization of the cytoskeleton, impacting cell morphology and migration, among other cellular processes. This interaction is therefore crucial for maintaining the normal functioning of cells and the integrity of tissues. Research has shown that CGN interacts with the RhoA activator GEF-H1 to suppress RhoA activity, thereby inhibiting cell proliferation, differentiation, and migration^[21–23]^. Accordingly, our present research shows that CGN upregulation underlies the inhibitory effect of polyphyllin H on the proliferation, migration and invasion of ovarian cancer cells.

The RhoA/Rock pathway plays an important role in the occurrence and development of tumors. RhoA is a small GTPase of the Rho family that activates the downstream Rho-associated protein kinases (ROCK1 and ROCK2) to regulate cell adhesion, motility, proliferation, and differentiation, thereby affecting the synthesis and secretion of inflammation-related molecules and producing corresponding biological effects^[21]^. The ROCK family of proteins encompasses a class of serine/threonine protein kinases that affect cell morphology, migration, and adhesion by exerting dynamic changes in the cytoskeleton. Activated ROCK proteins phosphorylate the myosin light chain, promoting acting-myosin cross-bridging and thereby enhancing cellular contraction. Overactivation of Rock kinases is especially important for the migration and invasion of tumor cells, allowing them to cross the basement membrane, enter the circulation, and metastasize to distant tissues and organs^[24,25]^. Moreover, evidence shows that RhoA/Rock pathway activation can increase tumor resistance and the generation of blood vessels^[26,27]^. Accordingly, inhibiting the activity of the RhoA/Rock pathway may lead to arrested tumor growth and metastasis, thereby improving clinical outcomes. Our research suggests that polyphyllin H may be used as a safe and effective RhoA/Rock2 pathway inhibitor, thereby making it an innovative and promising anti-ovarian cancer drug.

Ovarian cancer shows pathological heterogeneity. We found that polyphyllin H had therapeutic efficacy in the four PDX models examined, although to varying extents. By reviewing the expression levels of specific ovarian cancer markers in the different PDX models evaluated, we noted that compared to the respective control, the tumor inhibition rate and survival period in polyphyllin H-treated mice had greater significance in the Her2^+^ PDX model than in Her2^−^ PDX mice (Fig. 8N). This suggests that polyphyllin H treatment might be more meaningful for patients with HER^+^. Her2 is an important tumor marker, with significance for diagnosis, prognosis evaluation, and treatment of ovarian cancer^[28]^.Abnormal activation of Her2 can promote the proliferation, survival, and invasion of ovarian cancer cells through a series of downstream signaling pathways such as the PI3K/AKT/mTOR pathway^[29]^,facilitating rapid tumor progression linked to poor prognosis. Therefore, the fact that polyphyllin H showed greater efficacy in an Her2^+^ PDX model offers an enticing proof-of principle for its use in the treatment of Her2^+^ ovarian cancer, a tumor type associated with poor prognosis. Of course, it is noted that only four cases of PDX model were employed in the present study. We plan to substantially expand the cases of PDX models in future research endeavors to facilitate a more comprehensive and rigorous validation of this finding.

The administration of multiple drugs, often used in combination for cancer treatment, has deleterious effects on the tight junctions of intestinal epithelial cells, altering the colonization environment of intestinal flora. In this situation, the number of some conditional pathogenic bacteria may overgrow. This imbalance of the intestinal flora will cause diarrhea and may further aggravate intestinal inflammatory reactions, affecting the intestine’s normal digestive and absorptive functions^[30]^. When the intestinal absorption function is normal, it effectively absorb carbohydrate, protein, fat and other nutrients in food^[31]^. It is used to meet the energy needed for daily activities and metabolism, maintain normal body weight, and provide basal for the body anti-tumor^[32]^. Thus, gut dysbiosis related to anticancer treatment is a serious problem that needs to be urgently addressed. Desirably, the sequencing of the intestinal flora of CDX mice before and after polyphyllin H gavage treatment revealed no considerable changes in bacterial species. In addition, polyphyllin H gavage treatment also showed no damage to liver and kidney, bone marrow hematopoiesis function and major visceral histology. It is concluded that polyphyllin H might be well tolerated and may be a valuable adjuvant in cancer treatment.

## Conclusion

**Figure 9.**
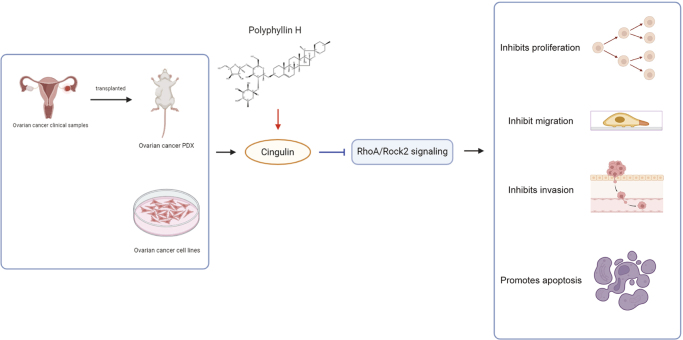
Schematic diagram.

In conclusion, this study demonstrates that polyphyllin H suppresses the tumor growth of ovarian cancer, inhibits the cell proliferation, invasion and migration, and promotes cell apoptosis via the CGN/RhoA/Rock2 pathway (Fig. 9). Moreover, the efficacy of polyphyllin H in inhibiting the malignant progression of ovarian cancer in PDX mouse, coupled with its favorable safety profile, highlights its translational potential for clinical applications.

## Data Availability

All data and materials are available for sharing if needed.

## Research registration unique identifying number (UIN)

Registration number for this study: ChiCTR2400080078 (http://www.chictr.org.cn/showproj.html?proj=213637).
